# Resequencing Three Candidate Genes for Major Depressive Disorder in a Dutch Cohort

**DOI:** 10.1371/journal.pone.0079921

**Published:** 2013-11-21

**Authors:** Eva C. Verbeek, Marianna R. Bevova, Zoltán Bochdanovits, Patrizia Rizzu, Ingrid M. C. Bakker, Tiny Uithuisje, Eco J. De Geus, Johannes H. Smit, Brenda W. Penninx, Dorret I. Boomsma, Witte J. G. Hoogendijk, Peter Heutink

**Affiliations:** 1 Department of Clinical Genetics, VU University Medical Center, Amsterdam, The Netherlands; 2 Department of Psychiatry, VU University Medical Center, Amsterdam, The Netherlands; 3 Department of Biological Psychology, VU University Medical Center, Amsterdam, The Netherlands; Yale University, United States of America

## Abstract

Major depressive disorder (MDD) is a psychiatric disorder, characterized by periods of low mood of more than two weeks, loss of interest in normally enjoyable activities and behavioral changes. MDD is a complex disorder and does not have a single genetic cause. In 2009 a genome wide association study (GWAS) was performed on the Dutch GAIN-MDD cohort. Many of the top signals of this GWAS mapped to a region spanning the gene *PCLO*, and the non-synonymous coding single nucleotide polymorphism (SNP) rs2522833 in the *PCLO* gene became genome wide significant after post-hoc analysis. We performed resequencing of *PCLO*, *GRM7*, and *SLC6A4* in 50 control samples from the GAIN-MDD cohort, to detect new genomic variants. Subsequently, we genotyped these variants in the entire GAIN-MDD cohort and performed association analysis to investigate if rs2522833 is the causal variant or simply in linkage disequilibrium with a more associated variant. *GRM7* and *SLC6A4* are both candidate genes for MDD from literature. We aimed to gather more evidence that rs2522833 is indeed the causal variant in the GAIN-MDD cohort or to find a previously undetected common variant in either *PCLO*, *GRM7*, or *SLC6A4* with a higher association in this cohort. After next generation sequencing and association analysis we excluded the possibility of an undetected common variant to be more associated. For neither *PCLO* nor *GRM7* we found a more associated variant. For *SLC6A4*, we found a new SNP that showed a lower P-value (P = 0.07) than in the GAIN-MDD GWAS (P = 0.09). However, no evidence for genome-wide significance was found. Although we did not take into account rare variants, we conclude that our results provide further support for the hypothesis that the non-synonymous coding SNP rs2522833 in the *PCLO* gene is indeed likely to be the causal variant in the GAIN-MDD cohort.

## Introduction

Major depressive disorder (MDD) is a psychiatric disorder that is characterized by persistent dysphoria, loss of interest and pleasure, changes in appetite and sleep, psychomotor retardation, feelings of guilt or worthlessness, inability to concentrate and recurrent thoughts of death or suicide [1]. Environmental circumstances have proven to influence the aetiology of the disease. It is more prevalent in women than in men and though MDD may develop at any age, the mean age of onset is 32 years of age, with a lifetime prevalence of 16.5%. Worldwide, MDD is one of the leading causes of disability [2]. The etiology of MDD is still largely an enigma, but stressful life events (SLEs) are a predictor for developing a depressive episode [3]. However, from twin studies it is known that heritability of MDD is approximately 40% [4].

In 2009, Sullivan et al. performed a GWAS for MDD on the Dutch GAIN-MDD cohort. Genome-wide significant association with MDD was not reached, but after post-hoc analysis including an Australian cohort the non-synonymous coding SNP rs2522833 in the gene *PCLO* showed nominal significance (P = 6.4E-8) [5]. The Perlegen chip used for this GWAS did not have full genome tagging capacity nor a gene-centered design, which is why we previously performed fine-mapping for seven genes that showed low P-values in the GAIN-MDD GWAS [6]. The increase of SNP coverage did not lead to the discovery of a more strongly associated variant. However, when combining the SNPs with the lowest P-value in *PCLO* with non-synonymous coding SNP rs2522833 in one haplotype, the P-value decreased, suggesting a possible undetected variant that is more strongly associated with MDD in the GAIN-MDD cohort [6]. In addition, in 2009 Bochdanovits et al. showed that either rs2522833 or an unknown variant that is in high LD with it, is most likely the causal variant in the GAIN-MDD cohort [7].

The non-synonymous coding SNP rs2522833 is a common variant with a minor allele frequency (m.a.f.) of 0.4. Since it is a common variant, we hypothesize that if this SNP is not the causal variant, the unknown variant that may be causal for the GAIN-MDD cohort will also be a common variant, as we expect this variant to be in high LD with rs2522833.

Besides the study of Sullivan et al., In literature there are other case-control studies replicating the role of PCLO in MDD [8], [9] Moreover, Minelli et al found that the *PCLO* gene was involved in personality traits that predispose to depression, showing a role of *PCLO* in MDD using endophenotypes [10].

As a follow-up study for the GAIN-MDD GWAS, the aim of this study is therefore to identify this common causal variant, by increasing the resolution of genotyping with next generation sequencing (NGS) followed by association analysis between the newly identified variants and MDD in the GAIN-MDD cohort.

To accomplish this, we sequenced 50 control samples from the GAIN-MDD cohort. Controls were used since we expect the undetected variant to be common and therefore also present in control samples. In addition, this will allow us to witness this variant against the background of the normal LD-structure of the Dutch population. Although we selected controls for sequencing, it was our aim to find the most associated variant within the cohort. Bochdanovits et al. in 2009 stated that either rs2522833 would be causal, or a variant in high LD with it. If homozygotes are selected for this variant rather than heterozygotes, it increases the possibility to detect other variants in high LD with the risk allele.

In addition to *PCLO*, which was selected based on our previous results, we also sequenced the genes *GRM7* and *SLC6A4*, which have been studied extensively as functional candidate genes for MDD in the literature. *GRM7* codes for the metabotropic glutamate receptor 7 and an intronic SNP in this gene showed a P-value that approximated genome-wide significance in a meta-analysis of three depression cohorts [11]–[13]. The *SLC6A4* gene codes for the serotonin transporter gene and plays an important role in the monoamine hypothesis of depression, according to which depressive phenotypes are caused by an imbalance in monoamines like serotonin. This gene has long been the topic of discussion, as there have been inconsistent results for the association with MDD of the promoter polymorphism in *SLC6A4* combined with SLEs. However, it has been shown that the polymorphism in the promoter in conjunction with SNPs within the gene itself and SLEs is more associated with depressive phenotype than just the promoter and SLEs [14].

## Methods and Materials

### Samples

The subjects for this study originated from two longitudinal studies, the Netherlands Study for Depression and Anxiety (http://www.nesda.nl) [15], designed to be representative of individuals with depression and/or anxiety disorders, and the Netherlands Twin Registry (http://www.tweelingenregister.org) for both of which sample collection and DNA isolation have been extensively described previously [5], [16].

50 control samples from the GAIN-MDD cohort were used for variant detection. Samples were selected based on their genotype for rs2522833 in the *PCLO* gene, with C being the risk allele, and consisted of 22 males and 28 females. Of the 22 males, 10 were CC and 12 AA. Of the females, 14 were CC and 14 AA.

For genotyping the detected variants and tag SNPs, we used the entire GAIN-MDD cohort, consisting of 1738 cases and 1802 controls, of which 1216 were male and 2324 female. All individuals had an age of 18–65 years and had self-reported western European ancestry. Ascertainment of cases was from outpatient specialist mental health facilities and by primary care screening. Inclusion criteria were a lifetime diagnosis of DSM-IV MDD as diagnosed by the Composite International Diagnostic Interview psychiatric interview, age 18–65 years, and self-reported western European ancestry.

Controls mainly came from the longitudinal cohort of the NTR. Longitudinal phenotyping includes assessment of depressive symptoms (via multiple instruments), anxiety, neuroticism and other personality measures. Inclusion required availability of both survey data and biological samples, no report of MDD at any measurement occasion, and low genetic liability for MDD. No report of MDD was determined by specific queries about medication use or whether the subject had ever sought treatment for depression symptoms and/or through the CIDI interview. Low genetic liability for MDD was determined by the use of a factor score derived from longitudinal measures of neuroticism, anxiety and depressive symptoms (mean 0, s.d. 0.7); controls were required never to have scored highly (≥0.65) on this factor score. Finally, controls and their parents were required to have been born in the Netherlands or western Europe. Only one control per family was selected.

### Ethical Issues

The NESDA and NTR studies were approved by the Central Ethics Committee on Research Involving Human Subjects of the VU University Medical Center, Amsterdam, an Institutional Review Board certified by the US Office of Human Research Protections (IRB number IRB-2991 under Federal-wide Assurance-3703; IRB/institute codes, NESDA 03-183; NTR 03-180). All subjects provided written informed consent. As part of the GAIN application process, consent forms were specifically re-reviewed for suitability for the deposit of deidentified phenotype and genotype data into the controlled-access dbGaP repository [17]. NESDA and NTR subjects were informed of participation in GAIN by means of newsletters.

### Gene Selection

The genes that were selected for targeted resequencing were *GRM7*, *PCLO*, *SLC6A4*. *PCLO* was selected based on the results from the GAIN-MDD GWAS and our previous fine mapping efforts, which suggested that either rs2522833 or an undetected variant in high LD with it would be the causal variant in the GAIN-MDD cohort. *GRM7* and *SLC6A4* were selected based on literature. *GRM7* codes for the metabotropic glutamate receptor 7 and was one of the top genes from a meta-analysis for MDD. *SLC6A4* encodes the serotonin transporter, which regulates serotonin availability in the synaptic cleft. The promoter contains a length polymorphism that is thought to modulate MDD in conjunction with SLEs [8]–[13].

### Library Construction

385 K NimbleGen Sequence Capture (Roche NimbleGen, Inc., Madison, WI, USA) arrays were custom-designed to capture the complete genomic locus of the *PCLO*, *GRM7* and *SLC6A4* genes, plus a 10 kb region upstream and downstream of the gene as defined by the UCSC Genome Browser B37 (http://genome.ucsc.edu) to capture possible regulatory regions as well. The total area consisted of 1,388,868 bp of which 1,217,056 bp was captured on the array (Table 1). A repeat mask was applied to reduce interference of genomic regions with a similar sequence.

**Table 1:** Base pairs and percentage of region covered on the Sequence Capture arrays.

Gene	Length(+/−10 kb)	Covered on array	% of region covered
*GRM7*	900.4 kb	793.3 kb	88.1
*PCLO*	478.9 kb	376.4 kb	87.8
*SLC6A4*	59.6 kb	47.4 kb	82.0

Sequence capture was performed according to the manufacturer’s protocol version 3.0 from December 2008, except for elution of DNA from the arrays, which was performed according to the manufacturer’s protocol for elution using sodium hydroxide version 1.0. After elution, arrays were cleaned using the NimbleGen Array Reuse Kit according to protocol. As a means of quality control, qPCR was performed for four control loci and enrichment was calculated.

At the time that this research was performed, the Roche Nimblegen and Illumina Solexa (Illumina, Inc., San Diego, CA, USA) platforms worked with different fragment sizes. In order to overcome this situation, an intermediate protocol was performed. Nimblegen fragments were ligated and then fragmented to the size corresponding to the Illumina protocols. For multiplexing purposes, index tags were ligated to the samples in order to identify them after sequencing.

### Sequencing

Sequencing was performed using the Illumina Solexa Genome Analyzer IIx (Illumina Inc., San Diego, CA, USA), by BaseClear (BaseClear BV, Leiden, The Netherlands). 50 base pair paired-end reads were generated in a multiplex fashion. Per lane 7 samples were loaded onto the flow cell, except for one lane that had 8 samples. As a control for the sequencing process, a PhiX DNA sample was also sequenced in a separate lane.

### Assembly and Variant Detection

The assembly of reads as well as variant calling was performed using CLC Bio Genomics Workbench (CLC Bio, Aarhus, Denmark). Full chromosome data (Build 37.1, hg19) was downloaded from NCBI and known variants were annotated using dbSNP131 from the UCSC Genome Browser. Reads were mapped back to the entire chromosome, allowing up to one mismatch and two unaligned nucleotides at the end of the reads or no mismatches and five unaligned nucleotides at the end of the reads.

Assembly was performed per sample.

SNP detection was performed for each sample individually. Minimum coverage was set at 20x and maximum coverage was set as the theoretical highest average coverage for that particular sample, by taking the number of reads × 50 (bp) and dividing that number by the length of the sequenced region. 35% of reads had to have an alternative allele in order to be called a heterozygous variant.

### Genotyping Procedure

All genotyping was performed using the Taqman OpenArray system (Life Technologies, Carlsbad, CA, USA), in accordance with the protocol of the manufacturer (version: 7/2010). 71 of the newly identified SNPs with unique sequences were the spotted onto the arrays. Of the newly detected SNPs with a m.a.f. ≥10%, 50 bp flanking sequences upstream and downstream were checked for similarities with other genomic regions using the BLAST tool [18].

In addition, 185 tag SNPs that were not previously genotyped in this cohort, were spotted on the arrays with m.a.f. 10% and r^2^ 0.9, tagging the genes +/−10 kb to include possible regulatory regions. For *GRM7* 47 new SNPs and 157 tag SNPs were genotyped, for *PCLO* 22 new SNPs and 27 tag SNPs, for *SLC6A4* 2 new SNPs and 1 tag SNP. The tag SNPs were selected by using Tagger software [19]. Arrays were designed to have 256 assays for 12 samples per array and were loaded using the OpenArray Accufill robot, manually loaded into a cassette and then PCR was performed in an NT cycler. After this, arrays were scanned with the OpenArray NT Imager. These SNPs were deposited at dbSNP.

The quality of scanned arrays was checked by visually assessing the location of the array in the scanner (the so-called Spotfind image) and ROX, VIC and FAM signals using ImageJ, (http://http://rsbweb.nih.gov/ij/). Genotypes for approximately 200 samples were analyzed simultaneously, using Taqman Genotyper Software v 1.0.1. This number of 200 samples was set by optimizing for clear clustering, without getting a bias due to too few data points. A home-made Perl script [20] was then used to combine all data and to create a pedigree file.

### Association Analysis

After the genotyping procedure, data was merged with genotyping data from the GAIN-MDD GWAS [5] and for *PCLO* with fine-mapping data [6]. We used the genome analysis tool PLINK to perform an association analysis [21]. We excluded samples with missing data >25%, SNPs with missing genotypes >10%, SNPs with m.a.f. <1% and HWE P-value <1E-05. A chi-squared test with one degree of freedom was used to perform the actual association analysis. A P-value of P = 5E-08 was considered to be genome-wide significant.

### Imputation

For imputation we used Beagle software [22]. 1000 genomes 2010-06 release CEU data was used as a reference [23], [24].

We did not use imputation data for the entire chromosome, as we were only interested in three genes and their regulatory regions. However, to leave the underlying LD-structure intact, we used a margin of 100 kb around each gene.

To extract the genes +/−100 kb from the full chromosome data of the 1000 genomes project, we used a home-made script written in Python [25]. Imputation was performed per gene with 100 Markov chain iterations, for all samples. All imputation was performed on the Lisa system cluster (www.sara.nl/systems/lisa).

### Gene-based Association

Since *PCLO* shows several sub-threshold association peaks in an 10E-06 magnitude, we also performed a gene-based association test and generated a single P-value for this gene rather than P-values for each SNP, by means of the VEGAS-tool [26]. VEGAS tests the evidence for association on a per-gene basis by summarizing the full set of markers and takes LD between markers into account by using simulation based on the LD structure of a set of reference individuals. We used our individual genotype data as a reference set, so that LD would be estimated specifically for the Dutch population. For each gene one million simulations were run.

### Epistasis Analysis

For each gene we performed an analysis of epistasis. Genes were classified into gene groups based on cellular function as determined by previous protein identification and data mining for synaptic genes and gene function, according to the method of Ruano et al. [27], in which synaptic genes are subdivided into 17 functional groups of genes on the basis of shared function into a biological process. We selected genes known to interact with, or be in the same functional gene group as either *GRM7* (G protein-coupled receptors), or *PCLO* (proteins involved in regulated secretion) or *SLC6A4* (ion and solute carriers and exchangers). Using the method of Lips et al. [28], genotypes for the SNPs existing in these genes were extracted from the GAIN-MDD GWAS data, after which epistasis analysis was performed with PLINK [21]. Table 2 depicts how many SNPs were tested for each gene.

**Table 2:** The number of SNPs used for the epistasis analysis.

Gene	Number of SNPs	Number of genes tested against	Number of SNPs tested against
*GRM7*	416	41	1220
*PCLO*	113	52	1579
*SLC6A4*	8	29	419

### Joint Reanalysis

We performed a joint reanalysis of 92 *PCLO* SNPs surrounding rs2522833 and rs2715148. For this analysis, we calculated Z-scores by performing logistic regression and dividing the slope for each data point by its standard error, similar to the method used by Sullivan et al. [5], [7] The absolute values of these Z-scores were then plotted against the square root of the r^2^ between one of these 92 SNPs with either rs2522833 or rs2715147.

## Results

### Sequencing

A total of 219 million reads were generated for all samples with 59 million reads mapping back to the region of interest (27%). All three genes reached an average coverage of at least 25 times and both *GRM7* and *SLC6A4* showed coverage of 20 times or higher for more than 50% of their base pairs. Using only basepairs with a minimum coverage of 20 times, we detected 4026 SNPs in total, of which 2658 were known previously in dbSNP131, 406 were found in the 1000 genomes 2010-06 release CEU data and 961 were newly discovered (Table 3).

**Table 3:** Coverage data and newly detected variants over all samples.

Gene	*GRM7*	*PCLO*	*SLC6A4*
Average Coverage	32.56	26.61	39.26
% bp covered ≥10x	75.47	61.63	78.98
% bp covered ≥20x	56.65	39.84	60.85
SNPs detected	2953	954	119
Exonic	8 (1 non-synonymous)	15 (4 non-synonymous)	0
Intronic	2893	885	78
UTR	5	15	9
In dbSNP	1923	659	76
In 1000 genomes project	374	29	3
Newly discovered	655	266	40
% SNPs with m.a.f. <5%	32.6	28.4	52.1
% SNPs with m.a.f. 5–10%	16.2	14.0	15.1
% SNPs with m.a.f. >10%	51.2	57.6	32.8

### Association Analysis

After variant detection, the GAIN-MDD cohort was genotyped for high resolution fine mapping using a tagging approach that included 71 newly discovered SNPs and 185 reported tag SNPs, as mentioned in methods and materials. The tag SNPs were selected so that all genes were covered 100% with m.a.f. >10% and r^2^ = 0.9, since the newly identified SNPs alone did not provide 100% coverage and we also aimed to recover the underlying LD-structure of the genes. For *GRM7* 47 new SNPs and 157 tag SNPs were genotyped, for *PCLO* 22 new SNPs and 27 tag SNPs and, for *SLC6A4* 2 new SNPs and 1 tag SNP. Several SNPs failed genotyping as the assays did not cluster very well, as they were either monomorphic or clusters were too close together to distinguish between genotypes. This lead to a total genotyping rate of 96.5% for SNPs. 293 samples (of which 60 cases and 233 controls) failed because of high levels of missing data, leaving a total of genotyping rate of 97.2% for samples.

After quality control, genotyping data was merged with SNPs from the GAIN-MDD GWAS and for *PCLO* also with SNPs from the fine mapping study that we performed previously [6], to add up to a total of 479 SNPs in three genes. After performing an association test with depression status as the dependent variable, the lowest P-value was found for in *PCLO* for rs2715147 at P = 1.5E-06 (OR = 0.79). For *GRM7* and *SLC6A4* the lowest P-values were P = 6.6E-05 (rs17664833, OR = 0.73) and P = 0.07 (SSNP38, OR = 1.18), respectively. For *GRM7*, the P-value was not lower than the lowest P-value in the GAIN-MDD GWAS. For *SLC6A4*, SSNP38 showed a lower P-value than the lowest in the GAIN-MDD GWAS (P = 0.09).

### Imputation

Since several SNPs were excluded from the analysis after quality control and several samples had missing genotypes, we imputed these missing genotypes using Beagle with the 1000 genomes CEU data as a reference panel for all missing genotypes.

**Figure 1:**
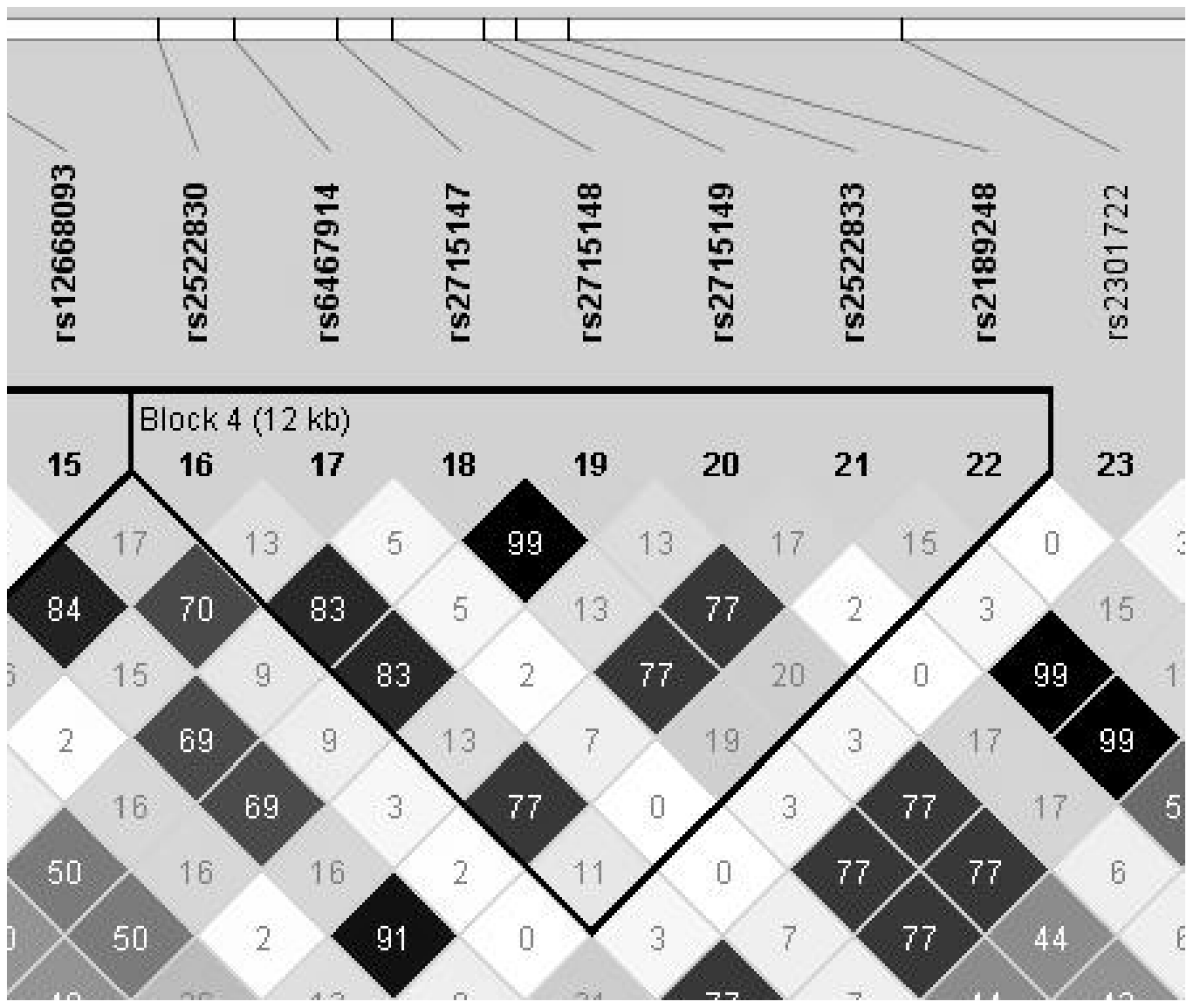
LD-plot of the region of interest in PCLO. The SNPs with the lowest P-values, rs2715147 and rs2715148 are in high LD with eachother and with rs2522833. This supports the hypothesis that either rs2522833 or a SNP in high LD with it is the most likely causal variant in this cohort.

We then again performed an association analysis. The lowest P-value was found for rs2715147 and rs2715148 at 2.3E-06 (OR = 0.80), located in the *PCLO* gene. These two SNPs are in strong LD with each other (r^2^ = 0.99) and with rs2522833 (r^2^ = 0.77); (Figure 1), in our data as well as in the 1000 genomes data and show a similar m.a.f. in the Dutch population when compared to the 1000 genomes CEU data. For *GRM7* and *SLC6A4* the lowest P-values were 2.61E-05 (rs17664833, OR = 0.71) and 0.08 (SSNP38, OR = 1.17), respectively.

These P-values did not provide a better association than the initial GAIN-MDD GWAS, and are therefore consistent with the hypothesis that rs2522833 may indeed be the causal variant in this cohort.

### Haplotypes

Using PLINK, we calculated the architecture of haplotype blocks for each gene, for the genotype data completed with imputed data. The lowest P-value was found for *PCLO* at P = 1.19E-05 (Table 4), showing no genome-wide significance. However, this block did not contain the SNPs that showed the lowest single SNP association (rs2715147 and rs2715148). When assessing the haplotype blocks in Haploview [29], we found that several SNPs surrounding rs2715147 and rs2715148 had an r^2^ lower than 0.2 and a single SNP P-value in the range of 0.5-0.1. Because of this lack of r^2^ and their high P-values, we created new haplotype blocks in which these SNPs were not included. Haplotype-based association analysis was performed again, which revealed the same block to have the lowest P-value. Nonetheless, this haplotype block does not show a better association with MDD than our single SNP association data. Moreover, the haplotype-based association test does not yield a P-value lower than rs2522833 in the GAIN-MDD GWAS.

**Table 4:** Haplotypes constructed using PLINK and their respective P-values.

Gene	SNPs in haplotype with lowest P-value	Lowest P-value
*GRM7*	rs3804925|rs17664792|rs17664833|rs779740|rs17047580	8.46E-05
*PCLO*	rs2371364|rs13237603|rs7810801|rs17210284|rs17282616|rs17156818	1.19E-05
*SLC6A4*	SSNP38|rs1042173	0.08

### Gene-based Association

Using the VEGAS tool, we generated P-values for all three genes by performing one million simulations. Since the human genome contains approximately 20,000 genes [30], we corrected for this number and considered 2.5E-06 (0.05/20,000) to be significant. Generating a gene-based P-value did not lead to a lower P-value result, since the lowest P-value was found for *PCLO* at P = 1.8E-05.

### Epistasis Analysis

For all three genes we performed an epistasis analysis in PLINK. First we tested all the SNPs that had P-values lower than 10E-05 in the single SNP association analysis. These SNPs yielded no P-values under 10E-04 in the epistasis analysis. Subsequently we tested all genotyped SNPs present in *GRM7, PCLO* and *SLC6A4*. The lowest P-value was found for *GRM7* rs1516569 in conjunction with rs9479791 (P = 3.8E-06), located in the intronic region of *OPRM1*, which codes for the Opioid Receptor Mu 1. For *PCLO* the lowest P-value was found at P = 9.4E-06 for rs17157173 together with rs16946196 in *DLGAP1*, which codes for Guanylate Kinase-Associated Protein (GKAP). The lowest P-value for *SLC6A4* (P = 2.42E-03) was found for rs4251417 with rs233112 in *DDAH1*, which regulates nitric oxide production. Since after correction for multiple testing, this epistasis analysis did not lead to a lower P-value than our single SNP analysis, we conclude that there is no evidence for an epistatic effect for SNPs of any of these genes with SNPs from interacting proteins. In addition, in literature, no effects of interaction between these genes have been described as yet.

### Joint Reanalysis

**Figure 2:**
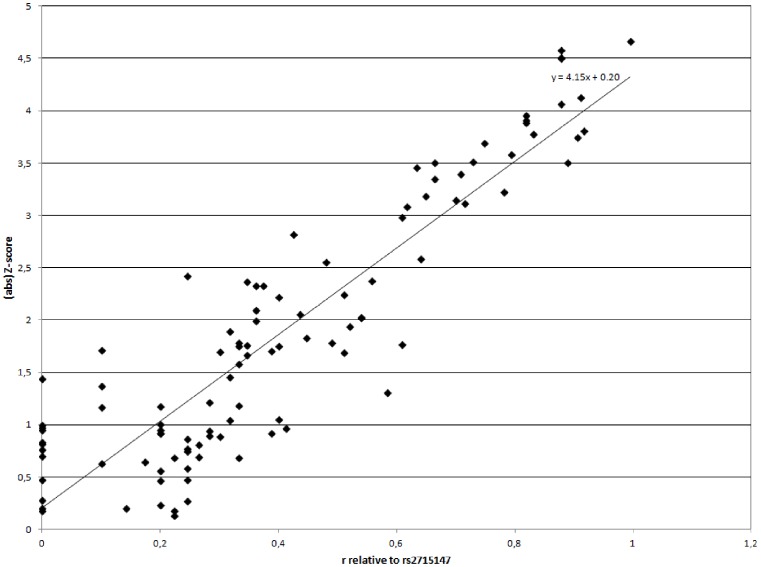
A joint re-analysis of 92 SNPs, in which Z-scores for each SNP are tested against the relative correlation of each SNP with rs2715147.

We then performed a joint reanalysis of 92 SNPs surrounding rs2522833 and rs2715147. The absolute values of Z-scores were plotted against the square root of the r^2^ between one of these 92 SNPs with either rs2522833 or rs2715147. When assuming the null-hypothesis of no association, one would expect that the slope of the linear fit would approximate 0, since SNPs in high LD with a causal variant will reflect the Z-score of this causal variant. When we assume that rs2522833 is the causal variant, the slope of the linear fit is 4.00, which increases slightly to 4.15 when assuming that rs2715147 is the causal variant (Figure 2).

## Discussion

For this study our aim was to detect all common variants in the genes *PCLO*, *GRM7* and *SLC6A4* in 50 control samples of the Dutch GAIN-MDD cohort and then genotype these variants for the full cohort, in order to test if we could identify a more likely causal variant than rs2522833 for MDD in this Dutch cohort.

Rs2522833 was the variant with the lowest P-value in the GAIN-MDD GWAS and the variant with the lowest P-value in our fine-mapping study (rs2715147) are both common variants in the Dutch population. Since we expect a causal variant to be in high LD with these SNPs and these SNPs are common, we would expect an undetected causal variant also to be common in our population, allowing control samples to be used. In addition, when using control samples, one can detect the underlying LD-structure of the common Dutch population, rather than a putatively skewed LD-structure in cases.

After genotyping newly identified SNPs and tag SNPs, several SNPs were excluded by our quality control. In order to acquire genotypes for all genotyped SNPs, we imputed using Beagle. Both before and after imputation, we did not find a stronger associated variant suggesting that rs2522833 may indeed be the causal variant in the GAIN-MDD GWAS. However, there may be several other reasons why we did not find a variant with a lower P-value than rs2522833.

First of all, in the Sullivan GWAS, rs2522833 only became nominally significant after post-hoc analysis with a cohort that used a similar method of ascertainment. This could imply that the sample size is too limited to detect variants with a small effect size. When looking at GWAS for other complex traits, successes mostly occur with a substantial larger sample size. This has already led to the discovery of new loci for example for Parkinson’s disease [31], multiple sclerosis [32] and breast cancer [33].

Previously, we investigated another cause for the apparent lack of associated variants: poor SNP coverage [6]. The array that was used for the GAIN-MDD GWAS, was a relatively early design and did not fully tag a substantial amount of the genome and was not designed in a gene-centered manner. This could lead to poor SNP coverage of certain genes, giving information only about the variants that have been genotyped for that gene in the GWAS and those variants that are in strong linkage disequilibrium (LD) with them. Therefore an associated variant that is not in LD with the genotyped variant, may go undetected. We tested whether an increase of SNP coverage may lead to a more associated variant, and though P-values slightly decreased, no genome-wide significance was found.

Thirdly, the phenotype ‘MDD’ may yet be too diffuse to find an associated variant. One way to solve this predicament is to create more specialized phenotypes, the so-called ‘endophenotypes’ that link together genetic factors and biological markers. There are many physiological steps to go from genetic variants to a psychiatric disorder, which is why psychiatry hopes to use the endophenotypes to move closer to the DNA level. A distinct endophenotype may increase effect size and therefore yield more significant results. In particular psychiatric disorders may benefit from endophenotypical descriptions, as their etiology is often complex and is thought to be a mixture of environmental and genetic causes [34]. However, this may be a laborious task, since the complexity of the disorder would lead to many different endophenotypes to investigate.

Additionally, if a single common variant only has a small effect size, one would expect epistasis to occur; several variants, which together cause an increase in risk. However, with the methodology of a GWAS or a case-control genotyping study, one will not easily detect all the variants involved in epistasis, exactly because of the small effect size. An alternative approach to this problem is to perform gene-based association tests, as genes are the functional units of the genome. For this, we used the VEGAS method, which tests the evidence for association on a per-gene basis by summarizing the full set of markers. It also takes LD between markers into account by using simulation based on the LD structure of a set of reference individuals. However, when taking all SNPs from a certain gene, a weight has to be assigned to each SNP, for which methods are still under debate. In addition, only part of the gene –i.e. a single domain- may be involved in the etiology of the disease. In this case taking the whole gene as a functional unit may cause a weaker association than when looking at the association with a specific domain [35], but the means to perform such tests are still limited. It may also be required for these tests to expand knowledge about the functions of protein domains in order to make a logical cut off which SNPs are to be included in a test. When more is known about the biological functions of various parts of the protein, one could for instance perform a joint re-analysis of SNPs located in specific domains, to increase the likelihood to find an association that has biological implications as well.

Also, we selected the newly detected variants that we genotyped based on a m.a.f. of more than 10% rather than on physical position, to increase the probability that the SNPs that we detected were actual variants instead of artifacts due to sequencing errors or contamination. It could well be that the variant(s) responsible for the pathology of MDD have a m.a.f. of less than 10% in our 50 control samples and therefore were not genotyped on the full GAIN-MDD cohort.

By sequencing 50 control samples we aimed to find a previously undetected common variant. The region between rs27175147 and rs2522833 has an average coverage of 25x. However, in the same region, on average 10% of base pairs had not been covered. This may explain why an additional variant was not found in this region. The lack of an associated common variant may also suggest that the “common disease, common variant” hypothesis may not hold true for either MDD or for this particular cohort. Since the beginning of the GWAS era, over 500 associated common variants have been found for a range of disorders. However, they usually only explain a small portion of the heritability and only account for a small increase in risk. An alternative scenario would encompass multiple rare variants with a m.a.f. of less than 5% to cause an increase in risk. To detect variants with an m.a.f. of 1–5%, at least 100 cases would have to be sequenced. With the per base costs of NGS lowering, it becomes more feasible to sequence larger groups, enabling the detection of multiple rare variants which may contribute to complex disorders [36], [37]. In the GAIN-MDD GWAS however, had rare variants been causal, there would not have been a marginally significant signal, unless if these rare variants would all have been recent and in the same haplotype. If these rare variants would cluster together in the same haplotype, then the variance explained by them should be so high, that they would have been expected to appear in linkage studies, which for MDD is not the case. Mixed models of both rare and common variants are currently under discussion, as it is indeed likely that complex disorders are under the influence of variants with various frequencies [38].

When taking all these factors into account, the fact remains that in this study as well as in three additional publications an identical area located in *PCLO* appears to contain the causal variant [5]–[7]. The area in which rs2715147, rs2715148 and rs2522833 are situated shows high r^2^ values, suggesting that the non-synonymous coding SNP rs2522833 or a SNP in high LD with it should be causal for the GAIN-MDD cohort. This SNP was found to be significant in the GWAS after post-hoc analysis with an Australian cohort, that used a similar method of ascertainment. The SNP changes a serine to an alanine in Piccolo’s calcium-binding C2A-domain. Overexpression of this C2A-domain causes a depression-like phenotype in mice [39], which makes the *PCLO* gene still an interesting candidate gene for MDD.

The selection of the three genes was based on previous results (*PCLO*) and on literature (*GRM7*, *SLC6A4*). The first gene we selected, *PCLO*, is situated on chromosome 7q11.23–q11.30. It encodes the protein Piccolo, which is located in the presynaptic active zone. These specialized areas of the presynaptic terminal have specific cytoskeletal properties to facilitate the preparation and release of vesicles into the synaptic cleft. In 2008, Leal-Ortiz et al. [40] showed that Piccolo is not essential for excitatory synapse formation, but it is a negative regulator of exocytosis, through modulation of Synapsin dynamics. This was later supported by Mukherjee et al. [41], who suggested that Piccolo and its highly homologous brother Bassoon function as tethering proteins that mediate efficient synaptic vesicle clustering. These observations make *PCLO* an interesting functional candidate for modulating the pathophysiology of MDD, as MDD is suggested to be caused by an imbalance in monoaminergic neurotransmission [42]. Besides the GWAS from Sullivan in 2009, a meta-analysis of three population-based studies also showed a genome-wide significant P-value for rs2522833, which further underscores a possible role for *PCLO* in MDD [9].

The second gene, *GRM7*, encodes the protein mGluR7. This is a metabotropic glutamate receptor, which mediates slowly modulating actions of glutamate on the release of neurotransmitters and the excitability of cells [43]. It is abundant in brain regions which are known to be critical in anxiolysis and antidepressant action, such as the amygdala and hippocampus. This suggests that mGluR7 is involved in the regulatory circuits that influence anxious and/or depressed behavior. In 2003, Cryan et al showed that *GRM7* ^−/−^ mice displayed less immobility following various stress paradigms. However, these anxiolytic/antidepressant results were still less pronounced than when animals were treated with anxiolytic drugs like benzodiazepines [11]. Furthermore, Mitsukawa et al., found an increase in glucocorticoid receptors in the hippocampus of *GRM7* ^−/−^ mice after stress paradigms. This connects mGluR7 to the hypothalamus-pituitary-adrenal axis (HPA-axis) which in turn is thought to be a key regulator in the stress response. In addition, *GRM7* ^−/−^ mice showed lower levels of the stress hormone corticosterone after stress paradigms than their *GRM7* ^+/+^ litter mates [12]. Moreover, in a meta-analysis of three studies for MDD, one of the strongest association peaks was observed for *GRM7* [43]. In summary, though GRM7 may be an eligible candidate gene in animal models, in this study we did not find evidence for a variant that showed a stronger association than in the GAIN-MDD GWAS.

And finally, *SLC6A4* encodes the serotonin transporter, which plays a pivotal role in the monoamine hypothesis of depression. The monoamine hypothesis states that depression is caused by the underactivity/imbalance of monoamines in the brain. The serotonin transporter regulates the availability of serotonin in the synaptic cleft by terminating the action of serotonin and recycling it in a sodium-dependent manner. Consequently, the serotonin transporter is a target for antidepressant drugs like selective serotonin reuptake inhibitors (SSRIs) which block the transporter and thereby increase available serotonin. *SLC6A4* has a length polymorphism in the promoter region, of which the short allele leads to less transcription of the gene. In 2003, Caspi et al. found a gene-environment interaction between the short allele and stressful life events as a predictor for MDD. However, replication efforts have been inconclusive and a meta-analysis in 2009 did not show this interaction [13], [44]. Although this length polymorphism may be associated with MDD in interaction with the environment in the cohort used by Caspi et al. and we found a slightly lower P-value for this gene, we do not find evidence for genome-wide association.

In conclusion, while in the 5 kb area between rs2715147 and rs2522833 in *PCLO* an average coverage of 25x was reached, we did not detect an additional common variant. Both in *GRM7* and *SLC6A4* previously undetected variants were found as well, but in neither genes we detected a variant that was more associated with MDD than rs2522833 in *PCLO*.

Although we cannot exclude the presence of multiple rare variants, our results suggest that, in accordance with the findings of Sullivan et al., non-synonymous coding SNP rs2522833 (or a variant in high LD with it) in *PCLO* gene is the causal variant responsible for the association peak in the GAIN-MDD cohort.
